# Supplementary material for: Are carbon emissions trading and green financial instruments synergistic? -Comprehensive quantitative research based on content analysis
Source: PLoS One. 2024 Mar 7;19(3):e0298601. doi: 10.1371/journal.pone.0298601 (PMC10919601; doi:10.1371/journal.pone.0298601)
Supplement: S1 Data — (ZIP) [file pone.0298601.s001.zip › Data/地级市Z_F工作报告.zip!/░▓╗╒/░▓╟∞╩╨/└┤╫╘╣1⁄2╓┌║┼í╛┬φ┐╦╩2╛▌═°í┐_2010.docx]

政府工作报告

——2010年1月20日在安庆市第十五届人民代表大会第二次会议上

市长 肖超英

　　各位代表：

　　现在，我代表市人民政府，向大会作政府工作报告，请予审议，并请市政协委员和其他列席人员提出意见。

　　一、2009年工作回顾

　　2009年是本届政府的开局之年，是新世纪以来经济社会发展最为困难的一年，也是我们积极应对危机挑战，追赶跨越、奋力崛起迈出坚实步伐的一年。全市人民在中共安庆市委的坚强领导下，深入学习实践科学发展观，全力做好保增长、保民生、保稳定的各项工作，市十五届人大一次会议确定的各项主要指标全面、超额完成。预计全市生产总值820亿元，同比增长13%，是近五年发展较快年份。财政一般预算收入96.24亿元，增长44.9%，增幅居全省第一位；全社会固定资产投资601.5亿元，增长37%；进出口总额4.98亿美元，下降7.02%，降幅低于全省15个百分点；社会消费品零售总额287.9亿元，增长19.1%；城镇居民人均可支配收入增长8.3%，农民人均纯收入增长10%；城镇登记失业率为4.2%，人口自然增长率为5％。，节能减排任务全面完成。全市经济企稳回升，民生明显改善，社会和谐稳定。

过去的一年，我们积极应对国际金融危机，保增长措施扎实有效。深入实施促进经济平稳较快增长的政策，相继出台保障就业、支持外贸出口、促进房地产和旅游消费措施。争取中央新增投资18.76亿元、地方政府债券转贷5.7亿元，带动地方投资增长。取消、停征、调整行政事业性收费项目42项，企业医疗、失业、工伤、生育保险费率降低30%以上，全市困难企业缓缴社会保险费11611万元，发放困难补助资金4300万元，减轻企业负担。市财政安排工业振兴基金3000万元、旅游发展资金800万元，促进产业发展。大力支持中小融资机构发展，典当企业增加到5家，信用担保公司达23家，小额贷款公司新增8家，中小企业担保基金和信贷风险补偿资金进一步充实。商标质押、股权出质、林权和动产抵押贷款等创新业务取得突破。强化金融服务，银企对接常态化，信贷投放增速与增量均创历史最好水平，新增贷款132.6亿元，有效缓解企业融资困难。

　  过去的一年，我们扎实推进结构调整，工业经济企稳向好。工业企业服务年活动扎实有效。规模以上工业增加值250.1亿元，增长25.3%。加强工业技改和重点项目建设，阿尔博白水泥、枞阳海螺四期、乙苯-苯乙烯、舒美特化纤一期建成投产，林浆纸一体化、华茂集团30万锭紧密纺、上峰水泥等工程进展顺利。规模以上企业增加179户,销售收入超十亿元企业9户、超亿元企业110户。建成中小企业创业基地31个，非公有制经济对经济增长的贡献率达到60%以上。加强与皖江城市带承接产业转移示范区规划的衔接，实施产业和空间布局规划，启动安庆化学工业区建设，省级开发区功能进一步完善，桐城双新开发区获省批准。与央企对接合作扎实推进。着力加强自主创新，国家级汽车零部件高新技术产业基地发展加快，石化产品质检中心建设进展顺利。与中科院合肥分院、中国电子科技集团38所合作加强。与安庆师范学院校企合作深入推进。国家知识产权试点工作取得成效。新增中国驰名商标2件。落实节能减排责任制，加大专项治理力度，化学需氧量、二氧化硫排放量大幅下降，城西污水处理厂和八县（市）污水处理厂全面建成。

　　过去的一年，我们持续加强“三农”工作，新农村建设稳步实施。全面落实强农惠农政策，“一卡通”发放涉农补贴12.1亿元。实施粮油棉高产创建行动，主要农产品产量创历史新高，粮食总产达270万吨。大力发展畜禽、水产规模化和标准化养殖，肉类禽蛋和水产品分别增产5%和3.9%。加快提升农业产业化，实施农业特色板块经济规划，市级以上龙头企业农产品加工产值增长19%。城郊型农业加快发展，大观蔬菜基地成为省级现代农业示范区。推进农村制度创新,集体林权制度主体改革顺利完成，农村集体土地确权发证工作全面启动。土地流转有序推进，种植业规模经营全省领先。农民经济合作组织发展到844户。农村金融改革取得突破，安庆独秀农村商业银行，桐城、岳西、宿松3家农村合作银行和江淮村镇银行挂牌营业。村民生产发展互助资金试点在国家扶贫工作重点县推开。农村基础设施建设进一步加强。完成20个乡镇总体规划、320个村庄建设规划编制。花凉亭水库大坝除险加固工程开工，135座病险水库除险加固完工。建成“村村通”公路1493公里,解决49.4万农村人口安全饮水问题，新建农村沼气池17751口。加强县域经济发展分类考核，推进扩权强县，实施扩权强镇试点。县域经济增幅高于全市平均水平2.1个百分点。

　　过去的一年，我们努力扩大有效需求，加速崛起动力持续增强。把投资和消费作为保持增长、化解危机的主要动力。狠抓项目带动，省“861”项目完成投资121.1亿元，市重点项目完成投资213亿元。济广高速岳潜段建成通车,望东长江公路大桥试桩工程开工。石化炼化一体化项目完成征地拆迁、规划、环评核准等各项工作，本月18日正式开工建设。南京至安庆城际铁路和安庆长江铁路大桥、东方造船基地、长风港一期工程加快建设。核电、阜景铁路、下浒山水库等项目前期工作扎实推进。强力推进招商引资,驻外招商有效开展。组织参加中博会、徽商大会等重大招商活动，举办皖台经贸洽谈会，赴绍兴、泉州、厦门、上海等地开展专题招商。实际利用市外资金482.6亿元，增长20.1%，利用境外资金突破2亿美元。安庆中西药业公司在境外上市,市城投公司发行企业债券12亿元。着力提升居民消费，巩固“万村千乡”和“新网工程”建设成果,积极推进家电、汽车和摩托车下乡,兑付补贴10896万元，家电下乡产品销售额居全省首位。举办房地产展示交易会，市财政安排住房交易税收补助700余万元，住房公积金贷款支持4573户居民购房,商品房销售面积和销售额分别增长122％和140%。与此同时,努力稳定外贸增长，壮大外贸队伍，优化出口商品结构,出口总额3.88亿美元，增长10.4%。

　　过去的一年，我们大力发展文化旅游，城市建设积极推进。高标准、有特色地举办第五届中国黄梅戏艺术节，展演16台黄梅戏新剧目，经贸活动成果丰硕，县（市）区节庆活动各具特色，黄梅戏影响力不断扩大，文化产业繁荣发展。非物质文化遗产保护得到加强。民族文化工作全省领先。强化文化基础设施建设，黄梅戏艺术中心建成运营，建成并开放中国黄梅戏博物馆，中国黄梅戏发展基地建设加快。加大文化旅游资源整合力度，陈独秀事迹陈列馆、五星级标准的安庆碧桂园凤凰酒店、七仙女国际大酒店相继建成，五千年文博园、孔雀东南飞文化园等文化旅游项目进展顺利。推进旅游经济强市建设，加强旅游工作目标管理绩效考核，政府主导、部门联动、群众参与的文化旅游发展格局初步形成。全年接待国内外游客1495.5万人次，实现旅游总收入98.1亿元，分别增长24.4%和25.9%，位居全省前列。积极推进“双百”城市建设，完成集贤北路、龙眠山路等进出口道路拓宽改造，城市基础设施进一步完善。加强城市美化、亮化和绿化，城市管理和文明创建工作力度加大，国家园林城市复检通过。文化名城、旅游城市和园林城市形象进一步提升。

　　过去的一年，我们着力保障改善民生，社会总体和谐稳定。把保障和改善民生作为保增长的出发点和落脚点，改革发展成果惠及人民。36项民生工程扎实有效，各级财政投入资金32亿元。加大促进就业力度，扎实推进就业再就业工作，城镇新增就业4.4万人。强化农民工培训与就业服务，积极引导返乡农民工就地就业，建成14个农民工创业园，第二批15个农民工创业园全面开工。扩大社会保险覆盖面，加强农民工、非公经济组织职工、城镇灵活就业人员参保工作，完善被征地农民养老保险制度，着手养老保险省级统筹基础工作，推进工伤保险、城镇医疗保险市级统筹,着力解决破产企业职工医疗保障问题。开工建设廉租住房37.2万平方米，10541户家庭享受廉租住房保障，实施8000多户农村危房改造。启动实施中小学校舍安全工程。扶贫开发力度加大，减少贫困人口8万人。深入推进义务教育均衡发展，市第一职教中心、技师学院建设和桐城师范升专工作进展顺利。市紧急救援中心、传染病医院投入运行，城乡公共卫生服务体系建设加强，甲型H1N1流感、手足口病防控卓有成效。全国听力语言康复事业规范建设试点和残疾人社区康复示范县创建工作扎实推进。出生人口性别比综合治理取得成效，计生家庭奖励扶助政策进一步落实，低生育水平保持稳定。有线电视网络数字化稳步推进。全民健身活动蓬勃开展，竞技体育水平明显提高。新一代天气雷达工程基本建成。完成第二次全国经济普查工作任务。“法治安庆”创建工作稳步实施，法律援助工作取得突破。扎实推进人民武装和“双拥”工作。老年人和妇女儿童权益得到保护。安全生产责任机制、联动机制进一步完善，总体形势好于往年。完善社会治安综合治理措施，打击和防范各类犯罪活动，重大节日、重大项目、重要活动安全保卫和信访维稳工作扎实有效。

　　过去的一年，我们深入学习实践科学发展观，政府自身建设得到加强。认真贯彻落实国务院关于加强市县政府依法行政的决定，开展依法行政推进年活动，落实行政执法责任制。启动实施政府机构改革,完善考核评议办法，公务员队伍建设得到加强。健全行政审批机制,优化窗口服务流程，办事效率进一步提高。自觉接受市人大及其常委会法律监督、市政协民主监督,广泛听取社会各界人士意见,积极办理人大代表议案和建议、政协委员建议案和提案,办复率达100%。认真回复网民问题，接受新闻舆论和群众监督。充分发挥“1584”等政风行风建设平台作用,深入推进政务公开,政府公信力不断提高。落实党风廉政建设责任制，深入推进惩治和预防腐败体系建设。认真落实中央厉行节约八项要求,压缩不必要支出，加强招投标采购管理，强化审计监督,扎实开展“小金库”和工程建设领域等突出问题专项治理，整治损害经济发展环境行为，纠正损害群众利益的不正之风。

　　在肯定成绩的同时，我们也清醒地看到，当前我市经济社会发展中还存在一些突出矛盾和问题：经济结构不够优，大项目大企业少，高新技术产业和战略性新兴产业基础薄弱；投融资渠道不够宽，上市融资没有突破；城乡收入差距依然较大，困难群众生产生活条件有待进一步改善；财政收入中县域税收比例偏低、市级地方收入偏低，支出压力加大；行政效能有待进一步提高，一些单位还存在办事推诿、拖拉扯皮现象。对此，我们将高度重视，采取措施，认真解决。

　　各位代表！在应对危机的复杂经济环境中，取得上述成绩，是省委、省政府和市委正确领导的结果，是市人大和市政协监督支持的结果，是全市人民共同奋斗的结果。在此，我代表市人民政府，向全市广大工人、农民、知识分子、干部、驻宜解放军指战员、武警、消防官兵和政法干警，向各民主党派、各人民团体和社会各界人士，致以崇高的敬意！向关心、支持安庆建设与发展的中央、省驻宜单位和海内外友好人士，向在我市创业的投资者、建设者，表示衷心的感谢！

　　二、2010年工作目标和主要任务

　　今年是“十一五”收官之年，谋划“十二五”关键之年。展望新的一年，我市经济发展面临着很多有利条件和机遇。综观宏观形势，世界经济呈现复苏，国内形势企稳向好，安徽崛起领先中部。宏观政策保持连续性和稳定性，增强针对性和灵活性，国家加大结构调整力度，政策支持更加注重加强“三农”、鼓励自主创新、促进消费、加大民生投入，我市追赶跨越的宏观环境更为利好。审视安庆发展，国家实施促进中部地区崛起规划，皖江城市带承接产业转移示范区建设全面启动，为我市又好又快发展创造了良好条件；作为皖江城市带三大产业组团之一、全省三大区域性中心城市之一，安庆发展跃上更高的战略平台，比较优势和后发优势日益凸显。尤其是一批打基础、管长远、增后劲的重大项目陆续开工建设，新的增长点正在加速形成。与此同时，也要认识到，当前国际金融危机余波未尽，国内外经济运行中不确定、不可预料因素依然存在，经济回升的基础还不牢固；区域竞争日趋激烈，我市经济增长的内生动力仍然不足，实现“千亿安庆”还需付出艰辛的努力。我们一定要切实增强紧迫感、责任感、使命感，审时度势，居高谋划，抢抓机遇，奋发有为，千方百计稳增长、转方式、调结构、促改革、惠民生，确保今年经济总量达到1000亿元，全面完成“十一五”规划的各项目标。

　　今年政府工作的总体要求是：以邓小平理论和“三个代表”重要思想为指导，深入贯彻落实科学发展观，加快推进发展方式转变，加快推进经济结构调整，加快推进改革开放，加快推进城乡统筹发展，加快推进和谐社会建设，全面完成“十一五”规划，确保实现“千亿安庆”目标，奋力夯实“十二五”发展基础。

　　综合考虑，初步安排今年经济社会发展的主要预期目标是：地区生产总值增长13%以上，财政一般预算收入增长13%，全社会固定资产投资增长25%，社会消费品零售总额增长19%，进出口总额增长10%，城镇居民人均可支配收入增长8%，农民人均纯收入增长8%，居民消费价格涨幅控制在5%以内，城镇登记失业率控制在4.5%以内，人口自然增长率控制在6.5％。以内，单位生产总值能耗降低3%，化学需氧量排放量降低1%。

　　为实现上述目标，要着力抓好以下九个方面工作：

　　（一）持续扩大有效投入。扎实开展“重点项目突破年”活动，以省“861”和市“3231”项目为重点，加快项目推进步伐，推动投资总量增长和结构优化。加快石化炼化一体化、林浆纸一体化、南京至安庆城际铁路和安庆长江铁路大桥、望东长江公路大桥及接线、文都和双岭500千伏输变电等重大项目建设。开工建设天柱山机场扩建、石门湖航道综合整治、潜望高速等项目。积极推进核电、北沿江高速、岳武高速、阜景铁路、下浒山水库、安庆长江二桥、安九路二期、桐城抽水蓄能电站等项目前期工作。加快中央投资项目建设，确保按期开工、如期建成。认真编制优势产业、新兴产业发展规划。着力谋划一批牵动性大、支撑力强、产业链长的重大项目，确保项目有效接续，促进结构调整和产业升级。加大项目争取力度，围绕政策导向，瞄准重点领域，力争更多项目列入国家和省投资计划。加强金融生态建设，充分发挥金融在资源配置中的积极作用。深化投融资体制改革，加强平台建设，拓宽投融资渠道。支持各县（市）区和市开发区培育企业上市，加强企业上市辅导与运作,新增1家上市公司。完善促进民间投资的具体措施，切实形成政府投资引导、社会投资主导的良性循环。

　　（二）着力调整产业结构。实施重点产业调整和振兴规划，新上一批十亿级项目，培育一批百亿级企业，着力打造石油化工、纺织服装、装备和机械制造等千亿产业。加快发展战略性新兴产业，支持鸿庆数控机床、盛运环保机械、飞凯高分子材料、光大光伏发电、和兴生物塑料等做大做强。加快中国安庆食品工业城建设。扶持壮大骨干企业，促进船柴、环新、曙光、丹凤、稼仙等企业发展，进一步增强支撑作用。全面深化与央企战略合作，推进首批合作项目落地建设，培育新的经济增长点。改善中小企业融资服务，鼓励支持全民创业，重点培育30个产业集群和20个产业集群专业镇。进一步完善和落实放宽市场准入、降低创业门槛、结构性减税、企业减负、信贷服务等方面优惠政策，促进非公有制经济快速发展。继续推进国有集体企业改革改制，加快资产重组和嫁接改造步伐，盘活存量。改革经营管理体制，引进战略投资者，加快纺织产业园和精品生产基地建设，放大增量，延长链条，做强华茂。

　　（三）加快转变发展方式。强化节能减排目标责任，抓好重点用能企业和重点领域节能减排，深入开展千家企业节能行动和节能产品惠民工程，加强办公和大型公共建筑节能监管，淘汰落后产能。探索发展低碳经济，推进低碳化改造和低碳技术应用，充分发挥森林固碳减排作用，走低碳化发展道路。加强支柱产业循环经济建设，加快大观循环经济产业园发展，提高资源综合利用率。依法集约利用土地，节约水资源，扩大太阳能、生物质能利用。严格执行建设项目环境影响评价制度，完善重点污染源在线监控措施，狠抓环境污染企业整改。完善产学研合作机制，加快组建石油化工、纺织服装、汽车零部件和农副产品加工产业产学研战略联盟。加快推进汽车零部件高新技术产业基地建设。创建石油化工、精细化工产业高新技术产业基地。发挥市科技创业服务中心作用，引导鸿庆、恒昌创建院士工作站，加速创新要素向企业聚集，不断增强自主创新能力和产业核心竞争力。加强知识产权保护，做好名牌产品培育、驰名商标申报和专利申请工作。坚持引资引智并重，引进各类创新创业人才，营造人尽其才、各尽所能的良好环境。

　　（四）坚持统筹城乡发展。进一步简政放权，落实县域发展扩权事项，加强对县域经济社会发展规划、重大基础设施、生态环境建设及重大突发事件处理等方面的指导协调，扶优扶强，加快发展，努力在全省争先进位。支持桐城加快融入合肥经济圈。毫不松懈地抓好“三农”工作。加快建设现代农业，创建现代农业示范区，提升农业产业化，培育一批年销售收入过20亿元的农业特色板块。落实强农惠农政策，抓好主要农产品生产供给，强化国家优质农产品基地建设，启动皖江农产品物流园区项目建设。稳定粮油棉生产，加强菜篮子基地建设。大力发展畜禽和水产养殖业。增强农业发展支撑能力，不断改善农业农村基础设施。加强耕地保护，推进农村土地整治和农田基本建设。实施病险水库除险加固工程，启动中小河流域治理，新增220个村通水泥路，解决38万人饮水安全问题。推进农村制度建设和创新，完善集体林权制度配套改革，积极引导农村土地规范流转，加快发展农民专业合作社。加大山区库区扶贫开发力度。完善“三农”金融服务体系，新增农村合作银行2-3家、村镇银行3-4家、农村资金互助社1家，小额贷款公司超过20家，引进1-2家股份制商业银行。鼓励和规范保险业及担保体系发展，稳步推进政策性农业保险。继续开展小城镇“宜城杯”竞赛活动。深入开展新农村建设试点示范。将新农村建设与城镇化相结合，努力在户籍制度改革、实现公共服务均等化等环节取得突破，进一步提高城镇化水平。拓展农民外出就业空间，促进农业转移人口逐步在城镇就业落户。重点支持县城和扩权强镇试点镇建设，坚持产业兴镇，培育主导产业，建设一批特色鲜明、功能齐全的重点城镇。开展城镇脏乱差专项整治，创建文明城镇，改善城乡面貌。

　　（五）积极承接产业转移。全面启动皖江城市带承接产业转移示范区规划建设，深化泛长三角区域分工合作，提升对外开放水平。实施“6+2”专项发展规划，主动出击，加强对接，尽快落实一批有牵动性的重大产业转移项目，为示范区建设开好头、起好步。大力加强承接平台建设，加强资源整合、促进优势互补，明晰功能定位、实现错位发展，不断增强园区承载力。编制铜池枞产业集中区总体规划和起步区控制性详规，推进安庆产业组团发展。加快各个省级开发区发展，全力推进安庆化学工业区建设，在承接产业转移竞争中抢占先机。完善招商引资绩效考核办法，加大市直单位招商力度，加强驻外招商，重点推进产业链招商，促进产业集群转移。以长三角为主攻方向，积极参加中博会、徽商大会等重大活动，精心组织开展系列专题招商活动。充分利用上海世博窗口宣传推介安庆。提高利用外资水平，力争在招大引强上取得突破。

　　（六）加快发展服务业。坚持生活性服务业与生产性服务业并重，推进传统服务业提升和现代服务业发展，增强服务业对发展经济、调整结构、改善民生的支撑力。加强城乡市场建设，不断优化消费环境，继续实施家电和汽车、摩托车下乡工程，积极引导市场流通、养老托幼、社区服务、医疗卫生等服务业发展，刺激居民消费增长。加强城市商务中心区建设，积极引进国内外大型知名零售企业，提升生活性服务业档次。完善房地产政策，加强房地产市场监管，加强住房公积金使用管理，做好低收入家庭住房困难保障工作，支持居民自住和改善型住房消费,保持房地产业健康稳定发展。支持迎江区开展城市商业改革发展示范区建设试点。认真落实促进外贸发展的各项政策，扩大机电、高新产品出口，壮大进出口队伍，培育加工贸易产业群，努力扩大进出口规模。做强光彩大市场，培育各类专业市场，扩大辐射范围，提升集散能力。围绕建设区域性物流枢纽，加快建设具备水陆联运、综合集散功能的物流园区，发展临港经济。加快发展现代物流、金融保险、信息咨询等服务业，引进培育大型物流企业，构建物流网络，加快发展生产性服务业。

　　（七）大力推进文化强市建设。加强黄梅戏文化挖掘、整理和研究，发挥黄梅戏基金会的作用，推进中国黄梅戏发展基地建设。实施黄梅戏精品工程，传承创新黄梅戏艺术。支持专业剧团开拓市场、下基层演出。加强黄梅戏学校建设。深化文化体制改革。推进五千年文博、汉玉创意、桐城玉雕等文化产业示范园区建设，培育一批文化龙头企业，构建区域特色文化产业群。加强历史文化资源的保护和利用，加快建设公共文化服务体系，让人民群众共享文化发展成果。进一步整合文化旅游资源，提升安庆旅游的文化内涵。探索文化旅游景区所有权、管理权与经营权分离，促进一体化管理、市场化经营。加强重点旅游项目建设，支持天柱山创建5A级旅游区和申报世界地质公园，推进花亭湖国家级森林湿地公园、龙山凤水文化旅游产业园建设，启动菱湖风景区创建4A级旅游区工作，开展民族宗教文化设施整体规划建设。加快望江武昌湖、枞阳浮山、宿松小孤山、岳西温泉等旅游景区和度假区建设。

　　（八）全力推进城市大建设。扎实开展“宜城崛起推进年”活动，深化市区财税体制改革，加快城区发展，推进区域性中心城市建设。完成土地利用和城市总体规划修编。建成“双百”城市基本框架。实施十大工程，全面启动政务新区基础设施建设，实施新河水系整治工程，加快东部新城建设步伐。以申办安徽省第十三届体育运动会为契机，以创建省级文明城市为目标，启动市、县体育设施建设。大力推进技师学院、安庆医专二期工程和市博物馆建设。加快市立医院新区建设和市第一人民医院迁建步伐。开展菱湖风景区东片和花亭大沟治理，实现雨污分流，逐步根除菱湖水污染的隐患。建成振风大道、顺安路和环城西路，开工建设机场大道、潜江路和文苑路。加大廉租房建设和危房改造力度，完成顺安路廉租住房和工人新村棚户区改造。建成马窝污水处理厂和生活垃圾焚烧发电厂，建设医疗废弃物集中处理场，启动城东污水处理厂二期工程。实施人民路灯饰、菱湖环湖亮化工程。完善天然气利用工程，迁建市第一自来水厂，确保安庆客运中心站建成投运。加大新河、石塘湖等水系污染防控力度，开展集贤关综合整治，限期关闭一些污染企业，着力解决空气、噪声污染等群众反映强烈的环境问题。抓好老城区小街小巷小区综合整治工作。

　　（九）切实保障改善民生。按照扩大内需和转变发展方式的要求,尽力而为、量力而行,今年民生工程扩大为42项，其中省定项目今年增加到33项，市级原定8项继续实施，新增加城区小街小巷路灯工程1项。落实促进就业再就业政策措施，做好高校毕业生、城镇失业人员和农民工就业工作，鼓励创业促进就业，加强培训优化就业，实施援助增加就业，统筹解决招工难与就业难的矛盾。加大社会保险扩面征缴工作力度，保障广大参保职工社会保险待遇。实施工伤保险、城镇居民医疗保险市级统筹，完善新型农村合作医疗和农村居民最低生活保障制度，加大对低收入群众的帮扶救助力度。加强社区建设，提高管理水平。优先发展教育事业，推动义务教育均衡发展,大力发展民办教育、职业教育和高等教育。积极推进中小学校舍安全工程,加快城乡义务教育学校标准化建设。推进基层医药卫生体制综合改革，全面落实当年医改任务，为人民群众提供安全、有效、方便、价廉的医疗卫生服务。健全计生利益导向机制，加强流动人口计生服务和管理，不断完善计生服务体系。扎实做好第六次全国人口普查工作。全面完成“五五”普法任务。全力维护社会稳定,积极推进社会矛盾化解、社会管理创新和公正廉洁执法三项重点工作。加强应急管理和突发事件处置，完善社会治安防控体系,打击和防范各类犯罪活动，提高社会公共安全保障能力。积极推进国防动员和后备力量建设,扎实做好“双拥”工作。进一步强化安全生产和安全监管的主体责任,保持安全生产形势稳定。加强信访工作,推进信访事项一站式服务和一揽子受理，依法维护群众合法利益。

　　三、切实加强政府自身建设

　　面对推进科学发展的新形势、新任务，我们必须认真贯彻党的十七届四中全会精神,落实“为民、务实、高效、清廉”的要求，保持良好的精神状态，不断改进政府工作，狠抓各项工作落实。

　　加强学习调研。坚持把学习调研作为提升工作水平的重要途径。扎实推进学习型政府建设，坚持用中国特色社会主义理论武装头脑，着力加强经济、政治、法律、文化、科技、社会和国际等各方面知识学习。在不断优化知识结构、开阔思路、把握规律中，提高战略思维、创新思维、辨证思维能力，增强领导经济工作的本领。加强调查研究，虚心向群众请教，集中民意，不断总结人民群众创造的新做法、新经验。要善于学习借鉴其他地区、部门、单位的好做法、好经验，紧密联系自身实际，有机地运用到实际工作中，丰富和拓展本地区、本部门、本单位改革发展的思路和办法。围绕宏观政策调整的着力点，针对新兴产业、改革创新、低碳经济、民生工程等系列重大课题，深入基层调查研究，不断提高解决实际问题的能力。扎实做好"十二五"规划编制工作，创新规划理念和方法，凝聚各方智慧和力量，切实增强规划的科学性、指导性和针对性。

　　坚持依法行政。坚持把依法行政作为工作的基本准则。完善政府常务会议学法制度，主动接受人大及其常委会的法律监督和人民政协的民主监督,认真接受社会公众、新闻媒体和网络舆论的监督。坚持科学民主决策，充分发挥咨询研究机构、专家学者、社会听证在决策过程中的作用，广泛听取方方面面意见。重大政策出台、重点项目实施和重要资金投向，一律集体研究决定，依法行使职权，履行职责。加强和改进行政执法监督、行政复议、规范性文件制定审理等工作。大力推进政务公开，完善新闻发布制度，扎实做好政府信息公开，切实提高政府工作透明度和公众参与度。推进依法决策制度建设，健全决策失误纠错改正机制和责任追究制度，开展政府工作绩效评估，着力营造公平、高效、有序的工作氛围。

　　力求务实高效。坚持把务实高效作为改进作风的不懈追求。牢固树立正确的政绩观，一切从实际出发，力戒急功近利、消极无为、官僚主义和形式主义，多办顺民意、解民忧、惠民生的实事。结合政府机构改革，精简行政审批事项，简化办事程序，清理行政性事业收费项目，推进效能提升。启动电子政务系统，推行网上审批，开展网络问政，创新联系群众方式，提高办事效率。大力整治文风会风，精简会议和文件。提倡开短会、讲短话、讲管用的话，力戒空话套话。完善年度目标考核制度，建立主要领导述效能、百名科长群众评长效机制。实施过程问责，推行阳光行政。健全有效保障落实的工作机制，对每项工作都要瞄准既定目标，把握时间节点，强化督查督办，奖优罚劣，推进落实。

　　保持勤政廉洁。坚持把勤政廉洁作为执政为民的自觉要求。提振精气神，保持好状态。加大治懒治散治庸力度，强化公务员队伍建设。保持干事创业、争创一流的激情，增强攻坚克难、志在必得的勇气，敢于超越、永不满足，说到做到、干就干好。完善政府重大投资项目公示制和责任追究制，加强投资控制和监管，规范招投标管理。加强对土地管理、工程建设、环境保护等重点领域的行政监察和审计监督，坚决纠正损害群众利益的不正之风。带头艰苦奋斗，厉行勤俭节约，加强惩治和预防腐败体系建设，认真落实党风廉政建设责任制，严厉打击腐败行为，坚决惩治腐败分子，维护政府良好形象。

　　各位代表！新形势赋予我们新使命，新任务引领我们新跨越。让我们高举中国特色社会主义伟大旗帜，在中共安庆市委的坚强领导下，紧紧团结和依靠全市人民，振奋精神，凝心聚力，顽强拼搏，扎实工作，为科学发展、加速崛起的美好明天而努力奋斗！
